# Better prognosis in surgical aortic valve replacement patients with lower red cell distribution width: A MIMIC-IV database study

**DOI:** 10.1371/journal.pone.0306258

**Published:** 2024-07-23

**Authors:** Liancheng Ruan, Lingxiao Zhu, Lang Su, Sheng Hu, Silin Wang, Qiang Guo, Bingen Wan, Shengyu Qiu, Yang Zhang, Yiping Wei

**Affiliations:** Department of Thoracic Surgery, The Second Affiliated Hospital of Jiangxi Medical College of Nanchang University, Nanchang, Jiangxi Province, China; University of Cape Town Faculty of Science, SOUTH AFRICA

## Abstract

**Background:**

Surgical aortic valve replacement (SAVR) currently stands as a primary surgical intervention for addressing aortic valve disease in patients. This retrospective study focused on the role of the red blood cell distribution width (RDW) in predicting adverse outcomes among SAVR patients.

**Methods:**

The subjects for this study were exclusively derived from the Medical Information Mart for Intensive Care database (MIMIC IV 2.0). Kaplan‒Meier (K-M) curves and Cox proportional hazards regression models were employed to assess the correlation between RDW, one-year mortality, and postoperative atrial fibrillation (POAF). The smooth-fitting curves were used to observe the relative risk (RR) of RDW in one-year mortality and POAF. Furthermore, time-dependent receiver operating characteristic (ROC) curves, the continuous-net reclassification index (NRI), and integrated discrimination improvement (IDI) were employed for comprehensive assessment of the prognostic value of RDW.

**Results:**

Analysis of RDW revealed a distinctive inverted U-shaped relationship with one-year mortality, while its association with POAF appeared nearly linear. Cox multiple regression models showed that RDW > 14.35%, along with preoperative potassium concentration and perioperative red blood cell transfusion, were significantly linked to one-year mortality (K-M curves, log-rank P < 0.01). Additionally, RDW was associated with both POAF and prolonged hospital stays (P < 0.05). There was no significant difference in length of stay in ICU. Notably, the inclusion of RDW in the predictive models substantially enhanced its performance. This was evidenced by the time-dependent ROC curve (AUC = 0.829), NRI (P< 0.05), IDI (P< 0.05), and K-M curves (log-rank P< 0.01).

**Conclusions:**

RDW serves as a robust prognostic indicator for SAVR patients, offering a novel means of anticipating adverse postoperative events.

## Introduction

Most patients with aortic valve disease, particularly aortic stenosis (AS), ultimately require aortic valve replacement [1]. AS is characterized by progressive calcification of the aortic valve and ranks as the most prevalent heart valve ailment in industrialized nations [2]. Surgical aortic valve replacement (SAVR) represents a surgical approach for treating AS.

Compared to transcatheter aortic valve implantation (TAVI), SAVR entails higher surgical risks and rates of postoperative complications. Among patients undergoing SAVR, the onset of postoperative atrial fibrillation (POAF) closely correlates with adverse outcomes. Kohno et al.[3] reported that POAF heightens the risk of hospital readmission, stroke, and mortality. Early identification and prevention of adverse events are paramount to improving patient prognosis. Imaging modalities such as echocardiography [4–6], electrocardiogram (ECG), computed tomography angiography (CTA) [7], and magnetic resonance imaging (MRI) [8] play critical roles in assessing cardiac function, while serum biomarkers such as cardiac troponins exhibit excellent sensitivity in detecting myocardial injury [9, 10]. However, their ability to accurately predict prognosis in SAVR patients is limited.

Red blood cell distribution width (RDW) is a common indicator reflecting variations in red blood cell size and volume. In recent years, the role of RDW in cardiovascular diseases has gained recognition [11]. Advanced age, Black race, and deficiencies in nutrients such as iron, folate, or vitamin B12 may potentially influence RDW levels [12]. Several studies have implicated RDW in various diseases, including chronic obstructive pulmonary disease (COPD) [13], atrial fibrillation (AF) [14–16], COVID-19 [17], and various cancers [18–20]. Some research has also demonstrated its predictive value in TAVI patients [15]. However, the role of RDW in SAVR patients remains unclear.

The Medical Information Mart for Intensive Care IV version 2.0 (MIMIC-IV v2.0) database [21, 22], a high-quality publicly restricted access critical care database, harbors extensive clinical data and has significantly contributed to influential studies [23–25]. This study aims to extract data from MIMIC-IV database to scrutinize the correlation between RDW and SAVR prognosis and to investigate the potential of RDW as a prognostic indicator for adverse outcomes.

## Materials and methods

### Database introduction

The MIMIC-IV v2.0 database [21], sourced from Beth Israel Deaconess Medical Center (BIDMC), offers a comprehensive collection of critical care data. It has been instrumental in driving extensive research in clinical informatics, machine learning, and epidemiology. This database represents an updated iteration of MIMIC-III, incorporating contemporary data and enhancing numerous aspects of the original dataset. One of the authors, LC Ruan, obtained access to this database with certification ID 50152346 and Name ID 11330672.

### Ethics statement

The human-related data used in this study were sourced from the publicly available MIMIC-IV database, which received approval from the Institutional Review BIDMC (Boston, MA) and Massachusetts Institute of Technology (MIT, Cambridge, MA). As outlined in their ethical statement, due to the project’s non-influence on clinical care and the de-identification of all protected health information, the requirement for individual patient consent was waived.

### Criteria for inclusion and exclusion

Data extracted in the study: (1) Patient records of individuals who underwent SAVR treatment were retrieved from the database using PostgreSQL Structured Query Language version 14.0 (PSQL v 14.0); (2) Adult patients aged 18 years or older were considered for inclusion; (3) Patients had to be in sinus rhythm preoperatively and not have experienced atrial fibrillation.

The exclusion criteria: (1) Patients with any laboratory data showing highly abnormal outliers, defined as values exceeding the upper quartile by 1.5 times the interquartile range (IQR) or falling below the lower quartile by 1.5 times IQR; (2) Missing RDW data; (3) Patients who have had procedures other than SAVR; (4) Patients with blood system diseases or tumors affecting RDW level.

### Data extraction

The database has independent identifiers that allow investigators to identify individual patient information. All date-related content in the database has been de-identified to protect patient confidentiality.

The MIMIC-IV v2.0 database includes data from patients admitted solely for SAVR for the first time. Population information and laboratory data within 48 hours prior to surgery were extracted, including: (1) Demographic information: age, gender, and ethnicity; (2) Vital signs: blood oxygen saturation via pulse oximetry (SpO2), heart rate, respiratory rate, systolic blood pressure (SBP), diastolic blood pressure (DBP); (3) Basic laboratory parameters: RDW, platelets, hematocrit, hemoglobin, potassium, white blood cells (WBC), creatinine, chloride, blood urea nitrogen (BUN), bicarbonate, anion gap; (4) Coagulation parameters: prothrombin time (PT), international normalized ratio (INR) used to assess the effectiveness of warfarin; (5) Concomitant diseases: renal disease, diabetes, rheumatic disease, chronic pulmonary, cerebrovascular disease, peripheral vascular, congestive heart failure, myocardial infarct; (6) Valve type: mechanical valve or bioprosthesis valve; (7) Perioperative red blood cell (RBC) transfusion; (8) Volume of chest tube drainage per admission; (9) Scoring systems: sequential organ failure assessment (SOFA) score and simplified acute physiology score II (SAPS II). Due to limitations in the database, we encountered challenges in obtaining data from the EUROSCORE II and STS scoring systems. Consequently, we incorporated the SOFA scoring and SAPS II scoring as alternatives in our study.

### Outcome definition

In this study, One-year all-cause mortality was defined as the primary endpoint. Secondary outcomes were POAF, length of hospital stays and length of ICU stays. The follow-up period spanned from initial admission for surgery to one year after final discharge or any recorded instances of death during the follow-up period. Prolonged length of stay (PLOS) was defined as a hospitalization (PLOS ≥ 9 days) or ICU (PLOS ≥ 3 days) stay exceeding its 75th percentile.

### Management of missing data

Some biochemical parameters were not available in the database. During parameter extraction, variables with missing values for more than 50% of the subjects were excluded directly [26]. For continuous variables with a missing value of less than 3%, the mean value is imputed, while for categorical variables, the median value is used to replace missing values.

### Statistical analysis

Mean ± standard deviation (SD) represented normally distributed variables, while median (the first and third quartiles) represented continuous variables with non-normal distributions. Differences between groups were assessed using t-tests or Wilcoxon rank-sum tests, based on the distribution characteristics of the data. Categorical data comparisons involved Fisher’s exact test, Chi-square test, and R×C contingency table chi-square test, with results presented as percentages. Youden’s index (sensitivity + specificity -1) [27] is the best single measure of effectiveness. An optimal Youden index, derived from the receiver operating characteristic (ROC) curve, determined the RDW level cutoffs to distinguish between high and low groups. Smooth fitting curves were utilized to investigate the linear correlation and relative risk (RR) between RDW and 1-year mortality of SAVR patients, as well as the association between RDW and POAF incidence in SAVR patients. The dissimilarities between the two groups in one-year mortality and POAF incidence were evaluated using the log-rank test and illustrated by Kaplan—Meier (K-M) curves. Univariate and multivariate Cox regression analyses were utilized to evaluate the hazard ratio (HR) of RDW for 1-year mortality. Multivariate regression identified statistically significant variables that were included in the construction of multiple Cox prognostic models. RDW is considered as a continuous variable and also as a categorical variable, allowing for comprehensive analysis of its impact on the study outcomes. Time-dependent ROC curves were used to adjust for outcome bias interfered with by time factors [28]. Furthermore, C index [29], integrated discrimination improvement (IDI) and continuous-net reclassification index (NRI) were employed to assess the net prognostic benefit of incorporating RDW [30] into the baseline model. A two-tailed hypothesis test was conducted at a significance level of p < 0.05. Statistical analyses were performed using Stata/MP 17.0 (StataCorp LLC, College Station, USA) and R software (Version 4.2.1) [31].

## Results

### Baseline characteristics

In accordance with the predefined exclusion and inclusion criteria (S1 Fig), a total of 630 SAVR patients were included. The optimal cut-off value for RDW determined by the Youden index was 0.489, corresponding to an RDW threshold of 14.35%. Based on this threshold, the population was divided into RDW above threshold group (> 14.35%, n = 498) and RDW below threshold group (≤ 14.35%, n = 132).

In the analysis of demographic characteristics, chest tube drainage volume per admission, aortic valve type, and in-hospital mortality, no statistically significant differences were observed between the RDW above threshold group and RDW below threshold group (Table 1). However, the former group exhibited significantly higher values in heart rate and several laboratory parameters, including platelet, creatinine, BUN, anion gap, compared to the latter group. Similar results were also observed in coagulation parameters and scoring systems. In terms of comorbidities, the group with RDW value below 14.35% group had a lower prevalence and a higher rate of perioperative RBC transfusion.

**Table 1:** Comparison of baseline characteristics of higher and lower RDW groups.

Variable	RDW≤14.35% (n = 498)	RDW>14.35% (n = 132)	P
**Demographics**			
Age (years)	67.0 (59.0, 73.0)	67.5 (57.0, 75.0)	0.657
Male, n (%)	339 (68.1%)	82 (62.1%)	0.197
Ethnicity, n (%)			0.139
White	399 (80.1%)	98 (74.2%)	
Black	19 (3.8%)	10 (7.6%)	
Other	80 (16.1%)	24 (18.2%)	
**Vital signs**			
Heart rate, beats/min	78.9 (73.6, 84.7)	80.4 (75.6, 88.1)	**0.032**
SBP, mmHg	112.1 (106.9, 117.1)	(104.8, 116.2)	0.236
DBP, mmHg	56.8 (52.9, 60.9)	57.3 (51.8, 63.1)	0.343
Respiratory rate, times/min	17.8 (16.2, 19.1)	17.9 (16.5, 20.0)	0.087
SpO2, %	97.6 (96.5, 98.6)	97.6 (96.6, 98.5)	0.728
**Laboratory events**			
WBC, 10^9^/L	11.4 (8.8, 15.0)	10.4 (7.5, 14.3)	0.062
RDW, %	13.1 (12.6, 13.5)	15.3 (14.7, 16.4)	**<0.001**
Platelet, 10^9^/L	129.0 (106.0, 157.0)	151.0 (113.0, 205.2)	**<0.001**
Hemoglobin, g/dL	9.5 (8.3, 10.6)	8.8 (7.8, 10.2)	**0.003**
Hematocrit, %	28.6 (25.2, 31.8)	27.6 (24.4, 31.5)	0.292
Potassium, mmol/L	4.3 (4.1, 4.5)	4.4 (4.1, 4.6)	0.241
Creatinine, mg/dl	0.8 (0.7, 1.0)	1.0 (0.8, 1.4)	**<0.001**
Chloride, mg/dl	105.3 (103.5, 107.0)	104.3(102.2, 106.9)	**0.005**
Bun, mg/dl	15.2 (12.7, 18.0)	18.7 (14.0, 26.1)	**<0.001**
Bicarbonate, mg/dl	23.2 (22.2, 24.8)	23.0 (21.9, 24.7)	0.140
Anion gap, mg/dl	12.0 (10.3, 13.7)	13.0 (11.5, 15.0)	**<0.001**
**Coagulation parameters**			
PT, s	14.49 (13.31, 17.07)	15.51 (13.69, 18.52)	**0.015**
INR	1.33 (1.23, 1.56)	1.43 (1.26, 1.71)	**0.041**
**Comorbidities**			
Renal Disease	51 (10.2%)	35 (26.5%)	**<0.001**
Diabetes	94 (18.9%)	39 (29.5%)	**0.008**
Rheumatic Disease	13 (2.6%)	11 (8.3%)	**0.002**
Chronic Pulmonary	90 (18.1%)	28 (21.2%)	0.411
Cerebrovascular Disease	30 (6.0%)	16 (12.1%)	**0.017**
Peripheral Vascular	113 (22.7%)	21 (15.9%)	0.090
Congestive Heart Failure	100 (20.1%)	61 (46.2%)	**<0.001**
Myocardial Infarct	34 (6.8%)	17 (12.9%)	**0.023**
**Valve type, n %**			0.057
Mechanical valve	122 (24.5%)	22 (16.7%)	
Bioprosthetic valve	376 (75.5%)	110 (83.3%)	
**Perioperative RBC transfusion**	355 (87.01%)	53 (12.99%)	**<0.001**
**Chest tube drainage, ml/admission**	315.00 (115.00, 950.00)	640.00 (342.50, 1652.50)	0.225
**Scoring systems**			
SOFA	5.0 (3.0, 7.0)	6.0 (4.0, 9.0)	**<0.001**
SAPSII	33.0 (27.0, 39.0)	37.0 (31.0, 43.0)	**<0.001**
**Outcome**			
In hospital mortality	2 (0.4%)	1 (0.8%)	0.507
1-Year mortality	6 (1.2%)	13 (9.8%)	**0.005**
POAF	89 (17.9%)	43 (32.6%)	**<0.001**
Days of stay in ICU	1.4 (1.2, 2.3)	1.6 (1.2, 3.3)	**0.002**
Days of stay in hospital	5.7 (4.8, 7.2)	6.7 (5.6, 13.1)	**<0.001**

BUN, blood urea nitrogen; DBP, diastolic blood pressure; INR, international normalized ratio; Other ethnicity, patients who are neither White nor Black; PT, prothrombin time; POAF, postoperative atrial fibrillation; RBC, red blood cell; RDW, red cell distribution width; SBP, Systolic blood pressure; SpO2, saturation of pulse oxygen; SOFA, Sequential Organ Failure Assessment; SAPS II, simplified acute physiology score II; WBC, white blood cell. Please refer to S2 Table for the complete diagnoses.

A detailed comparison of the same parameters between the one-year mortality group and the survival group after SAVR is provided in S1 Table. In S2 Table, significant differences in certain diagnostic outcomes are displayed based on RDW levels. Patients with RDW > 14.35% showed a higher incidence of acute and subacute infective endocarditis (6.28% vs. 0.00% in the RDW ≤ 14.35% group, p < 0.001), a lower prevalence of nonrheumatic aortic valve insufficiency (26.52% vs. 41.37%, p = 0.002), and a greater proportion of combined rheumatic disorders of mitral, aortic, and tricuspid valves (6.06% vs. 1.81%, p = 0.007).Among the outcome events, the RDW ≤ 14.35% group had lower 1-year mortality (1.2%) and POAF rates (17.9%) and had shorter hospital stays (5.7 days) or ICU duration (1.4 days) than the RDW >14.35% group.

### Cox univariate and multivariate regression

The results of Cox univariate and multivariate regression analyses are presented in Table 2 and S3 Table. In these analyses, RDW (OR = 3.72, 95%CI = 1.21, 11.37, p = 0.021), potassium levels (OR = 3.78, 95%CI = 1.04, 13.76, p = 0.043), and perioperative RBC transfusion (OR = 3.62, 95%CI = 1.09, 11.97, p = 0.035) were found to be statistically significant both in univariate and multivariate contexts.

**Table 2:** Univariate and multivariate Cox regression analyses for 1-year all-cause mortality.

	Unadjusted	*P*	Adjusted	*P*
	OR (95%CI)		OR (95%CI)	
**Demographics**				
Age (years)	0.99 (0.96, 1.02)	0.496		
Male, n (%)	0.67 (0.27, 1.67)	0.390		
Ethnicity, n (%)	0.69 (0.32, 1.49)	0.343		
**Vital signs**				
Heart rate, beats/min	1.02 (0.97, 1.07)	0.391		
SBP, mmHg	0.97 (0.91, 1.03)	0.314		
DBP, mmHg	1.00 (0.94, 1.07)	0.985		
Respiratory rate, times/min	1.11 (0.95, 1.30)	0.208		
SpO2, %	0.95 (0.69, 1.31)	0.760		
**Laboratory events**				
WBC, 10^9^/L	1.03 (0.95, 1.11)	0.480		
RDW, %	8.57 (3.26, 22.56)	**<0.001**	3.72 (1.21, 11.37)	**0.021**
Platelet, 10^9^/L	1.01 (1.00, 1.01)	**0.006**	1.00 (0.99, 1.00)	0.668
Hemoglobin, g/dL	0.79 (0.60, 1.05)	0.105		
Hematocrit, %	0.95 (0.86, 1.04)	0.260		
Potassium, mmol/L	4.21 (1.39, 12.71)	**0.011**	3.78 (1.04, 13.76)	**0.043**
Creatinine, mg/dl	1.53 (1.15, 2.02)	**0.003**	0.93 (0.61, 1.41)	0.729
Chloride/dl	0.83 (0.75, 0.92)	**<0.001**	0.92 (0.81, 1.04)	0.179
BUN, mg/dl	1.06 (1.03, 1.09)	**<0.001**	1.00 (0.95, 1.05)	0.912
Bicarbonate, mg/dl	1.09 (0.87, 1.36)	0.451		
Anion gap, mg/dl	1.04 (0.88, 1.24)	0.640		
**Coagulation parameters**				
PT, s	1.10 (1.0, 1.19)	**0.014**	0.69 (0.28, 1.68)	0.409
INR	4.25 (1.56, 11.56)	**0.005**	240.92(0.01,5711935)	0.286
**Comorbidities**				
Renal Disease	2.33 (0.84, 6.46)	0.105		
Diabetes	2.20 (0.87, 5.59)	0.097		
Rheumatic Disease	1.42 (0.19, 10.64)	0.733		
Chronic Pulmonary	1.59 (0.57, 4.41)	0.375		
Cerebrovascular Disease	0.70 (0.09, 5.24)	0.728		
Peripheral Vascular	1.01 (0.33, 3.04)	0.988		
Congestive Heart Failure	4.09 (1.64, 10.17)	**0.002**	1.76 (0.59, 5.24)	0.307
Myocardial Infarct	3.15 (1.05, 9.50)	**0.041**	2.17 (0.62, 7.60)	0.227
**Valve type**	2.56 (0.59, 11.06)	0.209		
**Perioperative RBC transfusion**	7.19 (2.39, 21.67)	**<0.001**	3.62 (1.09, 11.97)	**0.035**
**Chest tube drainage, ml/admission**	1.00 (1.00, 1.00)	0.804		
**Scoring systems**				
SOFA	1.11 (0.97, 1.28)	0.142		
SAPSII	1.03 (0.99, 1.06)	0.154		
**Diagnosis**				
Acute and subacute infective endocarditis	9.05 (2.09, 39.18)	**0.003**	2.89 (0.56, 15.03)	0.207

BUN, blood urea nitrogen; DBP, diastolic blood pressure; INR, international normalized ratio; OR, odds ratio; 95% CI, 95% confidence interval; PT, prothrombin time; POAF, postoperative atrial fibrillation; RBC, red blood cell; RDW, red cell distribution width (RDW is grouped based on a 14.35% threshold); SBP, Systolic blood pressure; SpO2, saturation of pulse oxygen; SOFA, Sequential Organ Failure Assessment; SAPS II, simplified acute physiology score II; WBC, white blood cell; Please refer to S3 Table for information on all diagnoses.

### Association with hospital and ICU length of stay

Table 1 indicated that there was no discernible difference in in-hospital mortality between the groups with RDW levels above and below the threshold of 14.35%. To mitigate bias, three models with varying degrees of adjustment were constructed, providing the risk of adverse outcomes in the RDW > 14.35% group compared to the RDW ≤ 14.35% group. In Model 1, no covariates were adjusted. In Model 2, covariates were adjusted for age, gender, and ethnicity. In Model 3, covariates were adjusted for age, gender, ethnicity, potassium levels, and perioperative red blood cell transfusion (Table 3). When analyzing RDW levels as a continuous variable, it was observed that in Model 1, the hazard ratio (HR) for ICU stay ≥ 3 days was 1.11 (95% CI: 1.02, 1.20, p = 0.015). This risk slightly decreased in Model 2 and Model 3, with HRs of 1.10 (95% CI: 1.01, 1.19, p = 0.026) and 1.03 (95% CI: 0.95, 1.13, p = 0.463), respectively. Similarly, for hospital stay ≥ 9 days, the HR in Model 1 was 1.21 (95% CI: 1.14, 1.29, p < 0.001), while in Model 2 and Model 3, it was 1.20 (95% CI: 1.13, 1.28, p < 0.001) and 1.14 (95% CI: 1.07, 1.22, p < 0.001), respectively. When considering RDW levels as a nominal variable, in Model 1, the HR for ICU stay ≥ 3 days was 1.51 (95% CI: 1.05, 2.17, p = 0.027), while in Model 2, it was 1.50 (95% CI: 1.04, 2.15, p = 0.030), and in Model 3, it was 1.12 (95% CI: 0.77, 1.64, p = 0.544). Regarding hospital stay ≥ 9 days, the HR in Model 1 was 2.63 (95% CI: 1.90, 3.65, p < 0.001), and in Model 2 and Model 3, it was 2.63 (95% CI: 1.89, 3.65, p < 0.001) and 1.97 (95% CI: 1.41, 2.77, p < 0.001), respectively. S4 Table indicates a significant association between patients experiencing POAF and ICU PLOS (p<0.001) as well as hospital PLOS (p<0.001).

**Table 3:** Predictive value of RDW level for adverse endpoints.

RDW level, %	Model 1		Model 2		Model 3	
	HR (95% CI)	p-Value	HR (95% CI)	p-Value	HR (95% CI)	p-Value
**Primary outcomes**						
**RDW as continuous variable**						
1-year all-cause mortality	1.31 (1.15, 1.50)	<0.001	1.35 (1.17, 1.55)	<0.001	1.25 (1.07, 1.45)	0.006
POAF	1.15 (1.07, 1.25)	<0.001	1.15 (1.06, 1.24)	0.001	1.09 (1.01, 1.20)	0.031
ICU stay ≥ 3 days	1.11 (1.02, 1.20)	0.015	1.10 (1.01, 1.19)	0.026	1.03 (0.95, 1.13)	0.463
Hospital stay ≥ 9 days	1.21 (1.14, 1.29)	<0.001	1.20 (1.13, 1.28)	<0.001	1.14 (1.07, 1.22)	<0.001
**RDW as nominal variable**						
1-year all-cause mortality	8.57 (3.26, 22.56)	<0.001	8.96 (3.40, 23.61)	<0.001	4.25 (1.35, 13.38)	0.013
POAF	1.98 (1.37, 2.88)	<0.001	1.99 (1.37, 2.89)	<0.001	1.57 (1.07, 2.32)	0.021
ICU stay ≥ 3 days	1.51 (1.05, 2.17)	0.027	1.50 (1.04, 2.15)	0.030	1.12 (0.77, 1.64)	0.544
Hospital stay ≥ 9 days	2.63 (1.90, 3.65)	<0.001	2.63 (1.89, 3.65)	<0.001	1.97 (1.41, 2.77)	<0.001

HR, hazard ratio; 95% CI, 95% confidence interval; POAF, postoperative atrial fibrillation; RDW, red cell distribution width.

Models 1, 2, and 3 were derived from Cox proportional hazards regression models.

Model 1 covariates were adjusted for nothing.
Model 2 covariates were adjusted for age, gender, and ethnicity.
Model 3 covariates were adjusted for age, gender, ethnicity, potassium, and Perioperative red blood cell transfusion

### RDW > 14.35% as a positive predictor of one-year mortality and POAF occurrence

**Fig 1:**
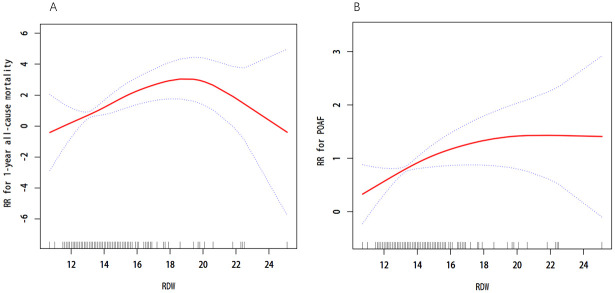
Fitting curves adjusted for variables with P < 0.05 in Cox multivariate regression. (A) Fitting curve of RDW and 1-year all-cause mortality in patients after SAVR. (B) Fitting curve of RDW and POAF in patients after SAVR. POAF, postoperative atrial fibrillation; RR, relative risk; RDW, red blood cell distribution width.

**Fig 2:**
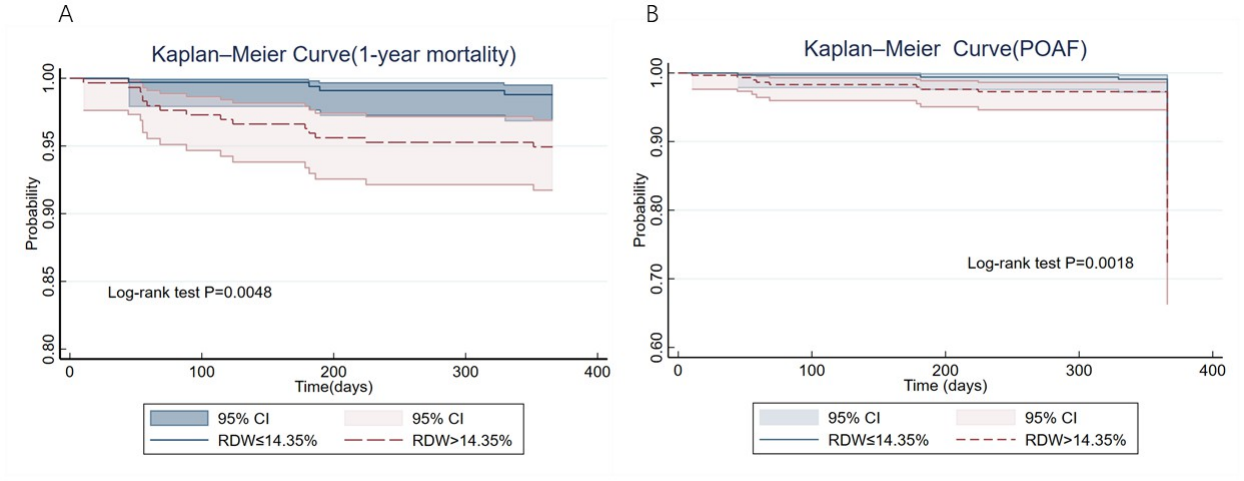
K-M curves of one-year survival and POAF in SAVR patients. (A) Kaplan—Meier survival curve of one-year all-cause mortality in patients after SAVR. (B) Kaplan—Meier survival curve of POAF in patients after SAVR. POAF, postoperative atrial fibrillation; RDW, red blood cell distribution width.

**Fig 3:**
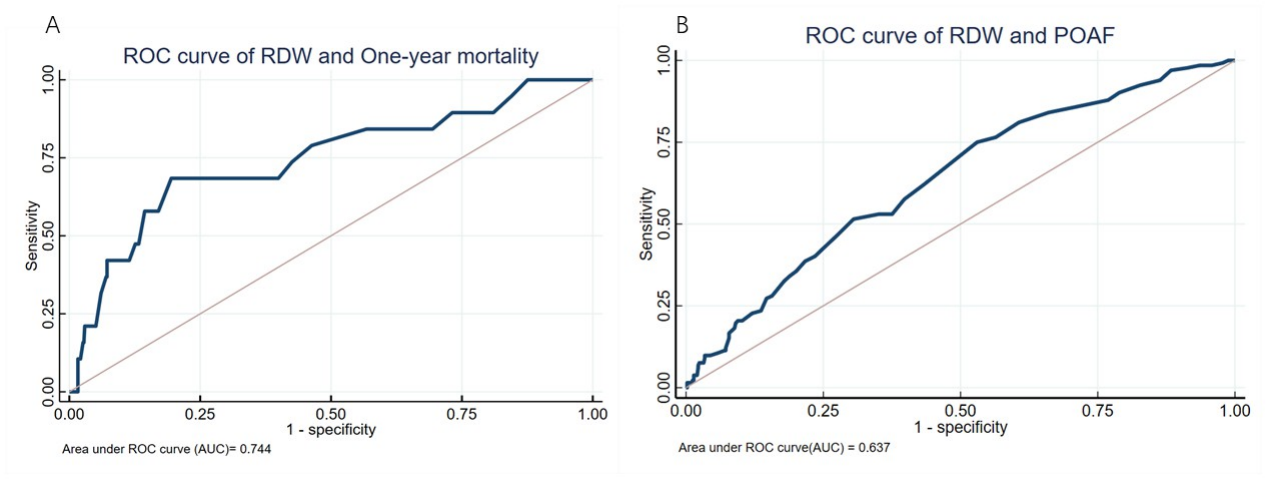
ROC curve in SAVR patients. (A) The ROC curve for predicting one-year mortality. The AUC = 0.744, 95%CI: 0.615–0.873. (B) The ROC curve for predicting POAF. The AUC = 0.637, 95%CI: 0.586–0.689. AUC, Area under the curve; CI, confidence interval; RDW, red blood cell distribution width; ROC, receiver operating characteristic curve; POAF, postoperative atrial fibrillation.

According to smooth curve fitting (Fig 1A and 1B), RDW showed an inverse U-shaped RR in one-year mortality and was nearly linear in POAF. However, when RDW levels exceeded 14.35%, there was a significant linear positive association with both POAF and one-year mortality. In Fig 1A, when RDW reached 18.60%, the RR of one-year mortality tended to decline, while in Fig 1B the RR consistently remained above 1. The K-M curves depicted in Fig 2A and 2B demonstrate that the RDW lower than 14.35% had significantly better survival outcomes and lower incidence of POAF compared with RDW above threshold level (p < 0.01 according to the log-rank test). The areas under the curve (AUC), showing the predictive value of RDW for one-year mortality and POAF in SAVR patients, prior to covariate adjustment, were 0.744 and 0.637, respectively (Fig 3A and 3B).

Based on the findings from Table 3, when nominal RDW was considered, the RDW > 14.35% group were positively associated with one-year mortality: Model 1: HR = 8.57 (95% CI, 3.26, 22.56, p < 0.001); Model 2: HR = 8.96 (95% CI, 3.40, 23.61, p < 0.001); Model 3: HR = 4.25 (95% CI, 1.35, 13.38, p = 0.013). Additionally, elevated RDW levels were identified as risk factors for POAF: Model 1: HR = 1.98 (95% CI, 1.37, 2.88, p < 0.001); Model 2: HR = 1.99 (95% CI, 1.37, 2.89, p < 0.001); Model 3: HR = 1.57 (95% CI, 1.07, 2.32, p = 0.021). When RDW was analyzed as a continuous variable, the RDW > 14.35% group continued to exhibit a positive correlation with one-year mortality: Model 1: HR = 1.31 (95% CI, 1.15, 1.50, p < 0.001); Model 2: HR = 1.35 (95% CI, 1.17, 1.55, p < 0.001); Model 3: HR = 1.25 (95% CI, 1.07, 1.45, p = 0.006). A similar trend was observed for the POAF outcome: Model 1: HR = 1.15 (95% CI, 1.07, 1.25, p < 0.001); Model 2: HR = 1.15 (95% CI, 1.06, 1.24, p = 0.001); Model 3: HR = 1.09 (95% CI, 1.01, 1.20, p = 0.031).

### Incremental value of RDW in predicting one-year survival

**Fig 4:**
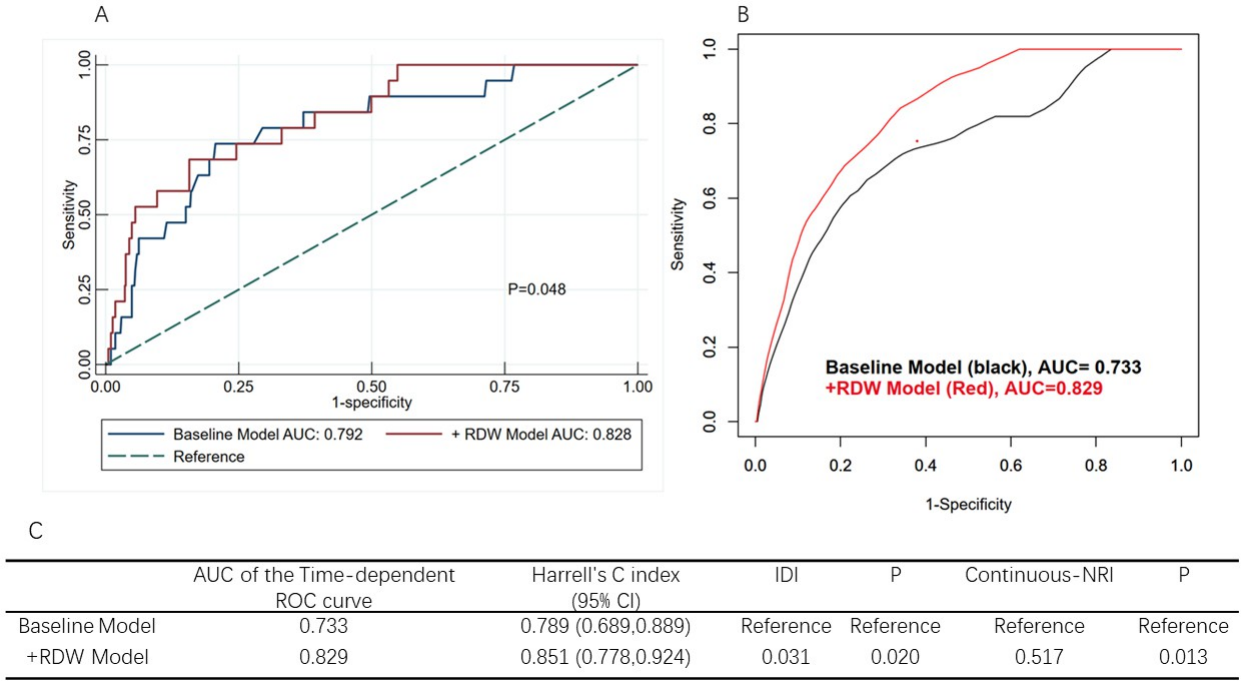
Comparative analysis of prediction models adjusted for cox multiple regression in one-year mortality prediction. (A) Comparison of ROC curves for two prediction models adjusted for Cox multiple regression in one-year mortality. Baseline model AUC = 0.792, +RDW model AUC = 0.828, P = 0.048. (B) Two time-dependent ROC curve models for one-year mortality adjusted for Cox multiple regression. Baseline model AUC = 0.733, +RDW model AUC = 0.829. (C) Harrell’s C index, IDI, and Continuous NRI were used to assess the predictive performance of the two models in Fig 4(B), all P < 0.05. AUC, area under the curve; ROC, receiver operator characteristic; RDW, red blood cell distribution width; Harrell’s C index, Harrell’s concordance index; 95% CI, 95% confidence interval; IDI, Integrated discrimination improvement; NRI, Net reclassification improvement; Baseline model includes variables that are significant in multivariate Cox proportional hazard analysis, including Potassium and Perioperative red blood cell transfusion; +RDW model is built on the basis of the baseline model with the addition of nominal RDW variable.

To assess the incremental predictive value of incorporating RDW into the baseline model for survival analysis, the time-dependent ROC curve, Harrell’s C index, continuous-NRI and IDI indices were employed. As depicted in Fig 4A, the inclusion of RDW increased the AUC from 0.792 (95% CI: 0.688, 0.896) to 0.828 (95% CI: 0.740, 0.916), with a significant difference between the two models (p = 0.048). To account for the effect of time factors on SAVR patients, time-dependent ROC curves were introduced (Fig 4B). In Fig 4C, the AUC for the baseline model and the +RDW model was equal to 0.733, 0.829 respectively. The Harrell’s C index values were 0.789 (95% CI: 0.689, 0.889) and 0.851 (95% CI: 0.778, 0.924). The IDI was 0.031 (P = 0.020), and the continuous-NRI was 0.517 (P = 0.013). These statistics suggest that the incorporation of RDW significantly enhances the prognostic accuracy for one-year mortality in patients undergoing SAVR compared to models without RDW.

## Discussion

This retrospective study identified the optimal preoperative RDW threshold as 14.35% using the Youden index and found that preoperative RDW > 14.35% served as a reliable predictor of adverse prognosis following SAVR treatment. This significance persisted even after adjusting for age, sex, race, and prognostic factors such as preoperative potassium concentration and perioperative RBC transfusion. Both non-time-dependent and time-dependent ROC curve analyses demonstrated a significant improvement in model predictive ability with the inclusion of RDW.

Anemia or decreased hematocrit is a common complication of many chronic diseases and affects RDW levels. Prolonged anemia exacerbates ventricular remodeling, particularly leading to left ventricular eccentric hypertrophy [32]. Anemia is prevalent among cardiac surgery patients, with reports indicating that over 45% of SAVR patients have preoperative anemia, while approximately 30% have it in TAVI [33]. Nagao et al. assessed patients with varying degrees of anemia due to AS and found that moderate to severe anemia significantly increased the risk of heart failure hospitalization or aortic valve disease-related death, regardless of SAVR or medical treatment [34]. Similarly, a prospective study by Arnold C T et al. involving 856 AS patients revealed a positive correlation between AS severity and anemia incidence, with anemia associated with higher all-cause mortality in AS patients. However, post-SAVR treatment, there was no significant difference in long-term survival between anemic and non-anemic patients, consistent with findings from our study [35]. The conflicting results may be attributed to the older age of patients included in studies by Nagao et al. (age > 74) and the higher severity of AS in those patients. Additionally, the relatively small number of patients reaching the mortality endpoint may lead to chance findings. Due to significant collinearity between hemoglobin and RDW, and the lack of significance in our study, hemoglobin was not adjusted for in predictive models to prevent overfitting and erroneous inferences.

Reasonable administration of blood products can mitigate the potential risks associated with anemia, whereas inappropriate transfusions may have adverse effects. Stephen D. et al. conducted a study involving 979 patients who underwent coronary artery or valve surgery at eight centers, revealing that perioperative RBC transfusion of 1U/2U increased the long-term mortality risk by 16% post-surgery [36]. Similarly, David W. and colleagues assessed 778 SAVR patients from the same institution, noting mortality only among those receiving more than 2 units of red blood cells, emphasizing the significance of perioperative blood conservation strategies in reducing transfusion rates[37]. This finding aligns with our results, indicating an elevated long-term mortality risk in SAVR patients with increased perioperative RBC transfusion. Transfusions of red blood cells may alter RDW values due to erythrocyte destruction, hemolytic reactions, and prolonged storage, highlighting their potential impact on patient outcomes [12]. To mitigate the potential influence of RBC transfusions, multivariate regression was employed to adjust for them, reaffirming a significant association between RDW and adverse outcomes. Thus, both RDW and RBC transfusions emerge as independent risk factors for poor prognosis in SAVR patients.

Despite potential limitations compared to traditional imaging techniques, RDW remains a valuable and cost-effective laboratory parameter for assessing erythrocyte volume heterogeneity [12]. Recent studies have identified RDW as a robust predictor of one-year mortality in high-risk elderly patients undergoing TAVI, underscoring its potential utility in risk stratification for this procedure [15]. While RDW has shown promise in risk prediction for TAVI patients, the applicability of these findings to individuals undergoing SAVR remains uncertain. Unlike TAVI, SAVR is associated with a higher incidence of perioperative complications, including massive bleeding, acute kidney injury, myocardial infarction, and new-onset atrial fibrillation [38]. Prior to this study, only the research conducted by Duchnowski et al. [39] had explored the potential association between elevated RDW levels and adverse outcomes in SAVR patients. However, this study had limitations, including a relatively small sample size and a failure to analyze RDW both categorically and continuously during data processing. Additionally, the association between elevated RDW levels and POAF was not determined, and the potential influence of RBC transfusions on postoperative outcomes was not addressed.

Zakkar et al. found that elevated levels of preoperative inflammatory markers and oxidative stress have been associated with the occurrence of POAF following cardiac surgery [40]. These markers [41], including C-reactive protein, TNF-α, IL-1β, IL-6, can be utilized to predict AF development. Inflammation not only decreases RBC survival rates, leading to greater variability in RBC volume within the bloodstream, but also disrupts iron metabolism and erythropoietin (EPO) production. This disruption contributes to a decrease in reticulocyte counts, causing erythroid maturation impairment and a subsequent reduction in the number of mature RBCs [42]. Additionally, TNF-α and IL-1 have been found to impede the production of EPO [12, 43], while IL-6 can induce inflammatory hyposideremia [44], promoting an elevation in the RDW value. Therefore, the link between RDW and POAF might be attributed to their shared association with inflammation and oxidative stress. This could potentially elucidate the higher incidence of POAF in SAVR patients with RDW>14.35% observed in our study. In line with Kubala et al.’s findings in SAVR patients, POAF was linked to higher long-term mortality rates compared to sinus rhythm patients [45]. Moreover, our study revealed a connection between POAF and extended ICU and hospital stays, potentially contributing to the significantly prolonged hospital stays observed in patients with RDW levels above the threshold.

Therefore, the full utilization of the efficiency of RDW can enable earlier detection of trends in POAF and prompt clinical intervention, significantly mitigating negative impacts on patients’ quality of life.

### Limitations of the study

Firstly, the exploration of the mechanisms underlying RDW changes in this retrospective study is limited to summarizing and inferring from previous research, with the true causal factors remaining unclear. Secondly, due to the study population being exclusively derived from the MIMIC-IV database and stringent inclusion criteria, the generalization of study findings to a broader population undergoing SAVR or different healthcare settings may be constrained. Although missing data is minimal (<3%), and the inferences drawn from the results remain robust, there is a possibility of a negative bias following rigorous imputation, such as non-significant hemoglobin levels in Cox univariate analysis. Furthermore, essential intraoperative variables crucial for risk assessment, including cardiopulmonary bypass, aortic clamp times, and unmeasured confounders like iron status, were not included in the study. Future research endeavors are warranted to corroborate these findings and delve deeper into the implications thereof.

## Conclusion

RDW is a valuable indicator for predicting short-term and long-term adverse events in patients undergoing SAVR. It is postulated that the pathogenesis of POAF may be mediated by inflammatory factors and oxidative stress, providing a novel potential prognostic indicator for SAVR patients.

## Supporting information

S1 Fig Flow chart of patient selection from the MIMIC IV v2.0 database. MIMIC, Medical Information Mart for Intensive Care database; SAVR, Surgical aortic valve replacement; RDW, red cell distribution width.
(TIFF)

S1 Table Comparison of baseline characteristics of survivors and dead patients in 1-year. BUN, blood urea nitrogen; DBP, diastolic blood pressure; INR, international normalized ratio; PT, prothrombin time; POAF, postoperative atrial fibrillation; RBC, red blood cell; RDW, red cell distribution width; SBP, Systolic blood pressure; SpO2, saturation of pulse oxygen; SOFA, Sequential Organ Failure Assessment; SAPS II, simplified acute physiology score II; WBC, white blood cell.
(DOCX)

S2 Table Comparison of diagnoses between the groups with RDW levels above and below the threshold of 14.35%. RDW, red cell distribution width.
(DOCX)

S3 Table Cox univariate and multivariate regression of patients diagnoses for 1-year all-cause mortality. NA: Not Available; OR, odds ratio; 95% CI, 95% confidence interval. The variables included in Cox multifactor regression were those with statistical significance in univariate regression (Table 2).
(DOCX)

S4 Table Relationship between POAF and adverse outcomes. POAF, postoperative atrial fibrillation.
(DOCX)
