# Supplementary figures and images for: Better prognosis in surgical aortic valve replacement patients with lower red cell distribution width: A MIMIC-IV database study
Source: PLoS One. 2024 Jul 23;19(7):e0306258. doi: 10.1371/journal.pone.0306258 (PMC11265686; doi:10.1371/journal.pone.0306258)

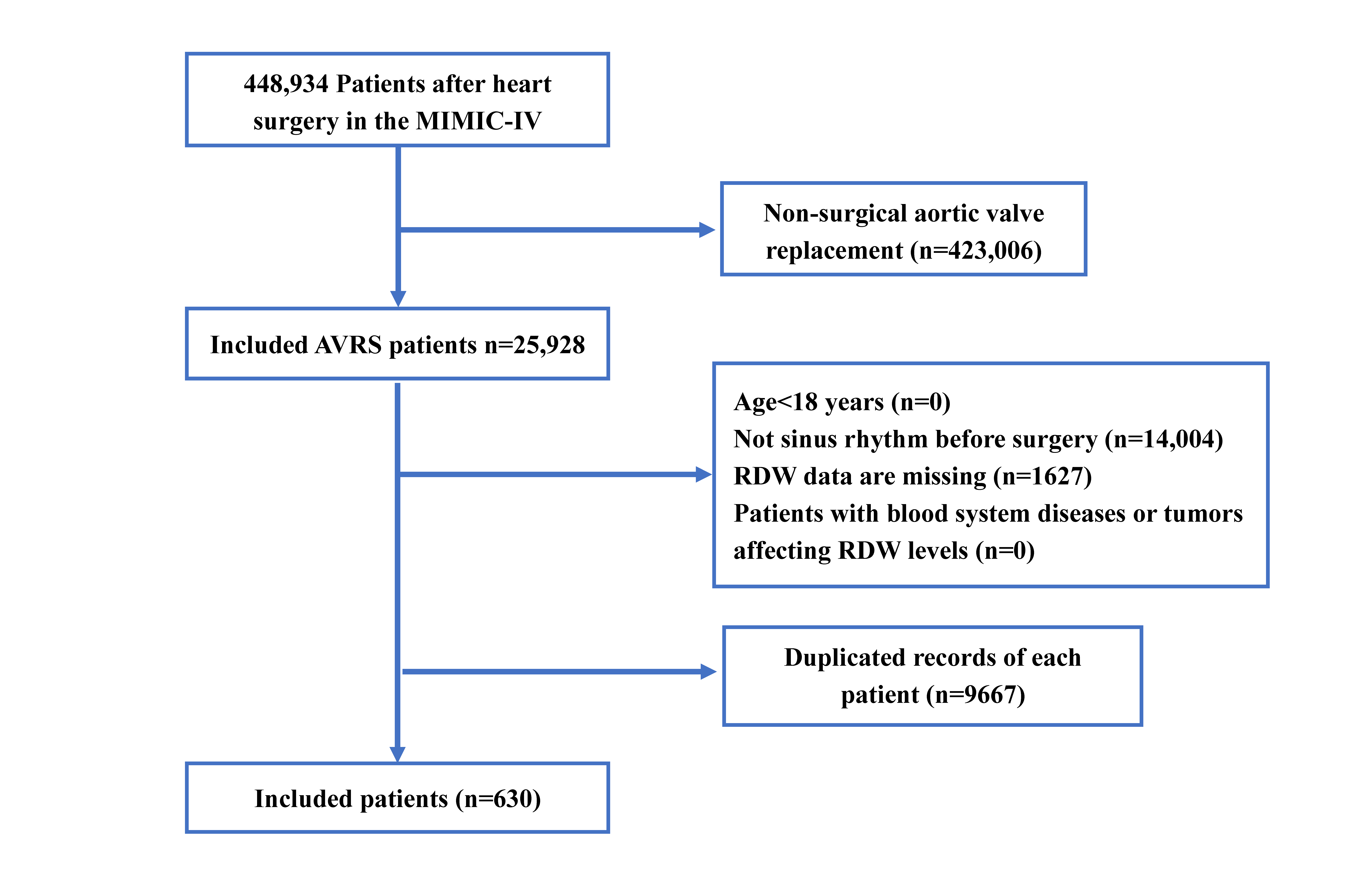

Supplement: S1 Fig — MIMIC, Medical Information Mart for Intensive Care database; SAVR, Surgical aortic valve replacement; RDW, red cell distribution width. (TIFF) [file pone.0306258.s001.tiff]
