# Supplementary material for: Better prognosis in surgical aortic valve replacement patients with lower red cell distribution width: A MIMIC-IV database study
Source: PLoS One. 2024 Jul 23;19(7):e0306258. doi: 10.1371/journal.pone.0306258 (PMC11265686; doi:10.1371/journal.pone.0306258)
Supplement: S1 Table — BUN, blood urea nitrogen; DBP, diastolic blood pressure; INR, international normalized ratio; PT, prothrombin time; POAF, postoperative atrial fibrillation; RBC, red blood cell; RDW, red cell distribution width; SBP, Systolic blood pressure; SpO2, saturation of pulse oxygen; SOFA, Sequential Organ Failure Assessment; SAPS II, simplified acute physiology score II; WBC, white blood cell. (DOCX) [file pone.0306258.s002.docx]

**S1 Table**

| **Variable** | **Survivors** | **Dead patients** | **P** |
| --- | --- | --- | --- |
| **Number of people, n** | (n=611) | (n=19) | <0.001 |
| **Demographics** |  |  |  |
| Age (years) | 67.00 (58.50, 74.00) | 68.00 (55.50, 74.00) | 0.595 |
| Male, n (%) | 410 (67.10%) | 11 (57.89%) | 0.401 |
| Ethnicity, n (%) |  |  | 0.448 |
| White | 480 (78.56%) | 17 (89.47%) |  |
| Black | 29 (4.75%) | 0 (0.00%) |  |
| Other | 102 (16.69%) | 2 (10.53%) |  |
| **Vital signs** |  |  |  |
| Heart rate, beats/min | 79.06 (73.85, 84.97) | 80.69 (77.92, 88.46) | 0.264 |
| SBP, mmHg | 112.04(106.69, 117.06) | 110.50(103.01, 115.69) | 0.348 |
| DBP, mmHg | 56.94 (52.77, 61.38) | 56.41 (53.24, 60.74) | 0.805 |
| Respiratory rate, times/min | 17.81 (16.27, 19.28) | 18.12 (16.60, 20.55) | 0.202 |
| SpO2, % | 97.62 (96.55, 98.53) | 98.00 (96.49, 98.64) | 0.945 |
| **Laboratory events** |  |  |  |
| WBC, 10^9^/L | 11.25 (8.60, 14.90) | 12.30 (8.60, 17.45) | 0.364 |
| RDW, % | 13.20 (12.70, 14.00) | 14.70 (13.35, 16.00) | **<0.001** |
| Platelet, 10^9^/L | 131.00 (107.00, 161.00) | 159.00 (131.00, 212.50) | **0.030** |
| Hemoglobin, g/dL | 9.40 (8.20, 10.60) | 8.30 (7.50, 9.75) | 0.092 |
| Hematocrit, % | 28.50 (25.00, 31.70) | 26.60 (24.15, 30.75) | 0.227 |
| Potassium, mmol/L | 4.32 (4.10, 4.54) | 4.57 (4.31, 4.74) | **0.008** |
| Creatinine, mg/dl | 0.86 (0.70, 1.06) | 1.00 (0.75, 1.44) | 0.064 |
| Chloride, mg/dl | 105.20(103.33,107.00) | 103.80 (100.94, 106.00) | **0.045** |
| Bun, mg/dl | 15.50 (13.00, 19.00) | 19.30 (14.36, 24.72) | 0.057 |
| Bicarbonate, mg/dl | 23.25 (22.00, 24.75) | 23.00 (22.19, 24.50) | 0.957 |
| Anion gap, mg/dl | 12.07 (10.60, 14.00) | 10.83 (10.00, 14.59) | 0.458 |
| **Anticoagulation situation** |  |  |  |
| PT, s | 14.60 (13.36, 17.30) | 17.04 (14.72, 21.21) | **0.014** |
| INR | 1.35 (1.23, 1.58) | 1.58 (1.37, 1.92) | **0.010** |
| **Comorbidities** |  |  |  |
| Renal Disease | 81 (13.26%) | 5 (26.32%) | 0.103 |
| Diabetes | 126 (20.62%) | 7 (36.84%) | 0.088 |
| Rheumatic Disease | 23 (3.76%) | 1 (5.26%) | 0.737 |
| Chronic Pulmonary | 113 (18.49%) | 5 (26.32%) | 0.389 |
| Cerebrovascular Disease | 45 (7.36%) | 1 (5.26%) | 0.729 |
| Peripheral Vascular | 130 (21.28%) | 4 (21.05%) | 0.981 |
| Congestive Heart Failure | 150 (24.55%) | 11 (57.89%) | **0.001** |
| Myocardial Infarct | 47 (7.69%) | 4 (21.05%) | **0.035** |
| **Valve type, n %** |  |  | 0.194 |
| Mechanical valves | 142 (23.24%) | 2 (10.53%) |  |
| Bioprosthetic valves | 469 (76.76%) | 17 (89.47%) |  |
| **Perioperative RBC transfusion** | 404 (66.12%) | 4 (21.05%) | **<0.001** |
| **Chest tube drainage, ml/admission** | 438.50  (172.50, 1130.00) | 737.50  (383.75, 1091.25) | 0.673 |
| **Scoring systems** |  |  |  |
| SOFA | 5.00 (4.00, 7.00) | 9.00 (5.00, 10.00) | 0.466 |
| SAPSII | 34.00 (27.00, 40.00) | 39.00 (35.50, 45.50) | **0.042** |
| **Outcome** |  |  |  |
| In hospital mortality | 0 (0.00%) | 3 (0.49%) | 0.759 |
| POAF | 121 (19.80%) | 11 (57.89%) | **<0.001** |
| ICU stay ≥ 3 days | 138 (22.59%) | 11 (57.89%) | **<0.001** |
| Hospital stay ≥ 9 days | 140 (22.91%) | 14 (73.68%) | **<0.001** |

BUN, blood urea nitrogen; DBP, diastolic blood pressure; INR, international normalized ratio; PT, prothrombin time; POAF, postoperative atrial fibrillation; RBC, red blood cell; RDW, red cell distribution width; SBP, Systolic blood pressure; SpO2, saturation of pulse oxygen; SOFA, Sequential Organ Failure Assessment; SAPSⅡ, simplified acute physiology score II; WBC, white blood cell.
