# Supplementary material for: Better prognosis in surgical aortic valve replacement patients with lower red cell distribution width: A MIMIC-IV database study
Source: PLoS One. 2024 Jul 23;19(7):e0306258. doi: 10.1371/journal.pone.0306258 (PMC11265686; doi:10.1371/journal.pone.0306258)
Supplement: S2 Table — RDW, red cell distribution width. (DOCX) [file pone.0306258.s003.docx]

**S2 Table**

| **Diagnoses, n (%)** | **RDW≤14.35%**  **n=498** | **RDW>14.35%**  **n=132** | **Total**  **n=630** | **P** |
| --- | --- | --- | --- | --- |
| Acute and subacute infective endocarditis | 0 (0.00%) | 9 (6.28%) | 9 (1.42%) | **<0.001** |
| Nonrheumatic aortic valve insufficiency | 206 (41.37%) | 35 (26.52%) | 241(38.25%) | **0.002** |
| Nonrheumatic aortic valve stenosis | 194 (38.96%) | 49 (37.12%) | 243 (38.57%) | 0.700 |
| Nonrheumatic aortic valve stenosis with insufficiency | 40 (8.03%) | 11 (8.33%) | 51 (8.11%) | 0.910 |
| Rheumatic Aortic valve insufficiency | 3 (0.60%) | 0 (0.00%) | 3(0.48%) | 0.371 |
| \| Rheumatic aortic valve stenosis \| \| --- \| | 1 (0.20%) | 0 (0.00%) | 1 (0.16%) | 0.606 |
| Combined rheumatic disorders of mitral, aortic and tricuspid valves | 9 (1.81%) | 8 (6.06%) | 17 (2.70%) | **0.007** |
| Rheumatic disorders of both aortic and tricuspid valves | 3 (0.60%) | 2 (1.52%) | 5 (0.79%) | 0.293 |
| Rheumatic disorders of both mitral and aortic valves | 42 (8.43%) | 18 (13.64%) | 60 (9.52%) | 0.070 |

RDW, red cell distribution width.
