# Supplementary material for: Better prognosis in surgical aortic valve replacement patients with lower red cell distribution width: A MIMIC-IV database study
Source: PLoS One. 2024 Jul 23;19(7):e0306258. doi: 10.1371/journal.pone.0306258 (PMC11265686; doi:10.1371/journal.pone.0306258)
Supplement: S3 Table — NA: Not Available; OR, odds ratio; 95% CI, 95% confidence interval. The variables included in Cox multifactor regression were those with statistical significance in univariate regression (Table 2). (DOCX) [file pone.0306258.s004.docx]

**S3 Table**

| **Diagnoses** | **Unadjusted** |  | **Adjusted** |  |
| --- | --- | --- | --- | --- |
|  | **OR (95%CI)** | **P** | **OR (95%CI)** | **P** |
| Acute and subacute infective endocarditis | 9.05 (2.09, 39.18) | **0.003** | 2.89 (0.56, 15.03) | 0.207 |
| Nonrheumatic aortic valve insufficiency | 1.18 (0.48, 2.94) | 0.720 |  |  |
| Nonrheumatic aortic valve stenosis | 0.42 (0.14, 1.26) | 0.121 |  |  |
| Nonrheumatic aortic valve stenosis with insufficiency | 1.34 (0.31, 5.78) | 0.699 |  |  |
| Rheumatic Aortic valve insufficiency | 0.00 (0.00, NA) | 1.000 |  |  |
| \| Rheumatic aortic valve stenosis \| \| --- \| | 0.00 (0.00, NA) | 1.000 |  |  |
| Combined rheumatic disorders of mitral, aortic and tricuspid valves | 0.00 (0.00, NA) | 1.000 |  |  |
| Rheumatic disorders of both aortic and tricuspid valves | 0.00 (0.00, NA) | 1.000 |  |  |
| Rheumatic disorders of both mitral and aortic valves | 1.82 (0.53, 6.24) | 0.342 |  |  |

NA: Not Available; OR, odds ratio; 95% CI, 95% confidence interval. The variables included in Cox multifactor regression were those with statistical significance in univariate regression (Table 2).
