# Supplementary material for: Better prognosis in surgical aortic valve replacement patients with lower red cell distribution width: A MIMIC-IV database study
Source: PLoS One. 2024 Jul 23;19(7):e0306258. doi: 10.1371/journal.pone.0306258 (PMC11265686; doi:10.1371/journal.pone.0306258)
Supplement: S4 Table — POAF, postoperative atrial fibrillation. (DOCX) [file pone.0306258.s005.docx]

**S4 Table**

|  | POAF | Non-POAF | P |
| --- | --- | --- | --- |
|  | N=132 | N=498 |  |
| In hospital mortality | 2 (1.52%) | 1 (0.20%) | 0.051 |
| ICU stay ≥ 3 days | 82 (62.12%) | 67(13.45%) | <0.001 |
| Hospital stay ≥ 9 days | 73 (55.30%) | 81 (16.27%) | <0.001 |

POAF, postoperative atrial fibrillation
